# The Histone Demethylase LSD1/ZNF217/CoREST Complex is a Major Restriction Factor of Epstein-Barr Virus Lytic Reactivation

**DOI:** 10.21203/rs.3.rs-5649616/v1

**Published:** 2025-01-13

**Authors:** Yifei Liao, Jinjie Yan, Isabella Kong, Zhixuan Li, Weiyue Ding, Sarah Clark, Lisa Giulino-Roth, Benjamin E Gewurz

**Affiliations:** 1 Division of Infectious Disease, Department of Medicine, Brigham and Women’s Hospital, Harvard Medical School, Boston, MA 02115, USA; 2 Center for Integrated Solutions to Infectious Diseases, Broad Institute of Harvard and MIT, Cambridge, MA 02142, USA; 3 Department of Microbiology, Harvard Medical School, Boston, MA 02115; 4 Harvard Program in Virology, Harvard Medical School, Boston, MA 02115; 5 Division of Pediatric Hematology/Oncology, Weill Cornell Medical College, New York, NY 10021, USA

**Keywords:** gamma-herpesvirus, lytic reactivation, epigenetic, histone demethylase, histone methyltransferase, lymphoma, lytic induction therapy, latency, DNA looping

## Abstract

Epstein-Barr virus (EBV) contributes to ~1.5% of human cancers, including lymphomas, gastric and nasopharyngeal carcinomas. In most of these, nearly 80 viral lytic genes are silenced by incompletely understood epigenetic mechanisms, precluding use of antiviral agents such as ganciclovir to treat the 200,000 EBV-associated cancers/year. To identify host factors critical for EBV latency, we performed a human genome-wide CRISPR-Cas9 screen in Burkitt B-cells. Top hits included the lysine-specific histone demethylase LSD1 and its co-repressors ZNF217 and CoREST. LSD1 removes histone 3 lysine 4 (H3K4) and histone 3 lysine 9 (H3K9) methylation marks to downmodulate chromatin activation. LSD1, ZNF217 or CoREST knockout triggered EBV reactivation, as did a LSD1 small molecule antagonist, whose effects were additive with histone deacetylase inhibition. LSD1 blockade reactivated EBV in Burkitt lymphoma, gastric carcinoma and nasopharyngeal carcinoma models, sensitized cells to ganciclovir cytotoxicity and induced EBV reactivation in murine xenografts. ZNF217 and LSD1 co-occupied the EBV immediate early gene BZLF1 promoter, which drives B-cell lytic cycle, as well as to the *oriLyt* enhancer regions recently implicated in EBV reactivation. LSD1 depletion increased levels of activating histone 3 lysine 4 (H3K4) methylation but not repressive histone lysine 9 methylation marks at BZLF1 and *oriLyt* and induced their interaction by long-range DNA looping. An orthogonal CRISPR screen highlighted a key H3K4 methyltransferase KMT2D role in driving EBV reactivation. Our results highlight H3K4 methylation as a major EBV lytic switch regulator and suggest novel therapeutic approaches.

## Introduction

Epstein-Barr virus (EBV) is a ubiquitous human gamma-herpesvirus that infects over 95% of adults and contributes to approximately 200,000 cancer cases per year worldwide^1^. EBV causes infectious mononucleosis and is associated with numerous human lymphomas, including endemic Burkitt lymphoma (BL), Hodgkin lymphoma, diffuse large B-cell lymphoma, primary central nervous system lymphoma, natural killer/T cell lymphoma and post-transplant lymphoproliferative disease^1, 2^. EBV also contributes to several epithelial malignancies, in particular nasopharyngeal carcinoma and gastric cancer^1–3^. EBV is also a major viral trigger of multiple sclerosis^4^.

EBV uses a series of latency programs to navigate the B-cell compartment and colonize the memory B-cell reservoir^5^. EBV latency programs utilize distinct combinations of Epstein-Barr nuclear antigens (EBNA), latent membrane proteins (LMP) and non-coding RNAs (ncRNAs) to alter B-cell activation and differentiation states^2, 6, 7^. In memory B-cells and in Burkitt lymphoma, EBV uses its latency I program, where EBNA1 is the only viral-encoded protein expressed. By incompletely understand epigenetic mechanisms, B-cell receptor cross-linking and plasma cell differentiation trigger EBV lytic reactivation, in which nearly 80 EBV proteins are de-repressed in a lytic cascade^8^.

Lytic reactivation is controlled at the level of two EBV immediate early genes, BZLF1 (also known as Z, Zta or ZEBRA) and BRLF1 (also known as R or RTA). Each encode viral transcription factors that drive expression of ~35 EBV early genes. BZLF1 and BRLF1 are each essential for EBV reactivation and induce each other’s expression^9, 10^. Their promoters are therefore heavily regulated, including by chromatin remodeling, DNA and histone modifications^11–13^. However, much remains to be learned about how EBV immediate early genes are epigenetically regulated in support of EBV latency, but in a manner that can be rapidly reversed upon receipt of lytic induction stimuli.

EBV early genes drive formation of nuclear replication compartments, in which linear EBV genomes are produced and serve as the template for EBV late gene expression^14–16^. EBV genomic *OriLyt* regions serve dual lytic cycle roles as viral genome origins of lytic DNA replication and also as enhancers for immediate early and late lytic gene expression^17–19^. Late genes encode virion structural components, including capsid proteins and the envelope glycoproteins. EBV glycoprotein 350 (gp350) binds to complement receptor 2/CD21 to mediate adhesion between infecting virions and target B-cells^20^.

Lytic genes are increasingly implicated in the formation and progression of EBV− associated cancers^21, 22^. Furthermore, multiple EBV latency oncogenes including LMP1 are expressed by the EBV lytic cycle^23–27^. Consequently, there is significant interest in defining how EBV latency is regulated, including to support EBV lytic induction therapeutic approaches, in which EBV reactivation sensitizes tumor cells to the antiviral ganciclovir^28–31^. Once activated, ganciclovir is highly toxic not only to lytic cells, but also to neighboring cells. Histone deacetylase (HDAC) inhibitors, DNA methyltransferase inhibitors, and additional small molecules induce varying degrees of EBV lytic reactivation^13, 32–34^. However, to be used clinically, lytic reactivation would need to be triggered in a larger fraction of tumor cells than is often achievable by available agents.

CRISPR genetic analysis highlighted MYC as a key repressor of EBV lytic reactivation in Burkitt cells^19^. MYC occupies EBV *oriLyt* region E-box sites to prevent long-range DNA looping between *oriLyt* and the *BZLF1* promoter region^19^. Cohesin, FACT, STAGA, and mediator complexes also promote EBV latency by supporting MYC expression and potentially also by supporting EBV genomic DNA looping^19^. Histone chaperones also play key roles in EBV latency, as knockout of the histone loader CAF1 or HIRA induced EBV lytic reactivation, as does depletion of SFPQ, which supports histone H1 expression^35, 36^. However, knowledge remains incomplete about how epigenetic mechanisms control the EBV lytic switch.

Here, we performed human genome-wide CRISPR-Cas9 screens for host factors critical for maintenance of EBV latency and also for factors that instead support lytic reactivation. These systematic CRISPR analyses highlighted key histone 3 lysine 4 (H3K4) methylation roles in support of EBV reactivation. The H3K4 demethylase LSD1 and its cofactors ZNF217 and CoREST were critical for EBV latency, whereas the H3K4 methyltransferase KMT2D instead supported EBV reactivation. ChIP-seq analyses highlighted that LSD1 and ZNF217 co-occupy EBV genomic *oriLyt* regions. CRISPR or chemical perturbation of LSD1 triggered higher order EBV genomic rearrangement that juxtaposed the *oriLyt* enhancers with the BZLF1 promoter region, triggered EBV lytic gene expression and sensitized Burkitt cells to ganciclovir cytotoxicity.

## Results

### CRISPR-Cas9 screen for host factors critical for EBV latency

**Figure 1.**
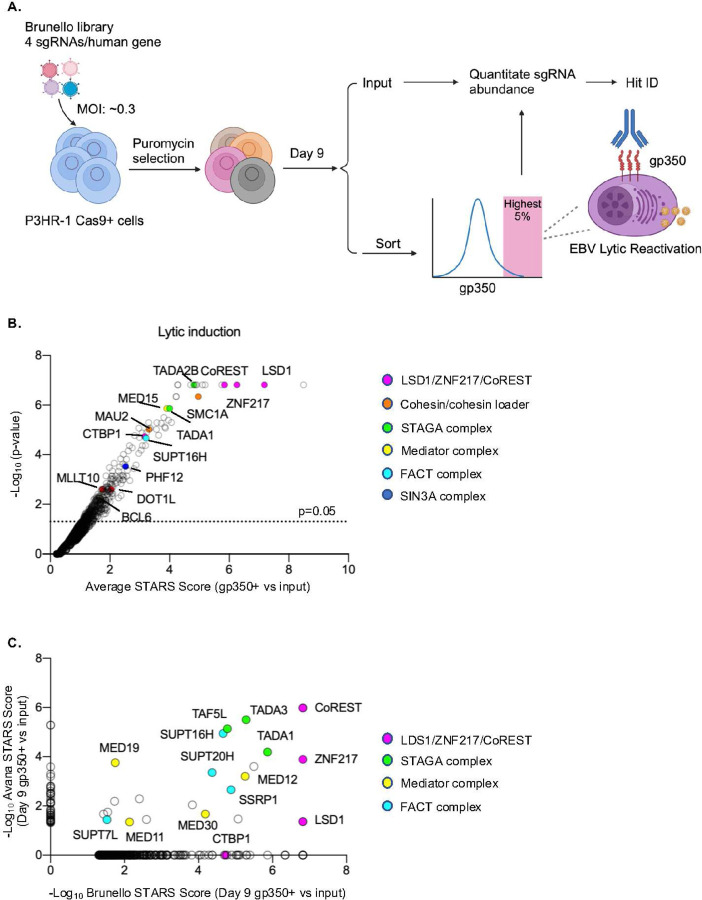
Human genome-wide CRISPR-Cas9 screen for host factors that restrict EBV lytic reactivation. (**A**) CRISPR-Cas9 screen workflow. Cas9+ P3HR-1 Burkitt B-cells were transduced with Brunello sgRNA library at multiple of infection (MOI) ~0.3. Nine days post-transduction, cells with de-repressed plasma membrane (PM) EBV lytic gp350 expression were sorted. sgRNA abundances in input vs sorted cells were quantitated and statistically significant hits were identified. (**B**) Volcano plots visualization of screen hits. Selected hits are highlighted by epigenetic category. (**C**) Cross-comparison of Brunello versus Avana sgRNA library CRISPR screens for host factors that repress EBV reactivation.

To gain insights into host factors required for EBV latency, we performed a human genome-wide CRISPR-Cas9 screen in P3HR-1 Burkitt cells, using the Brunello single guide (sgRNA) library^37^. Brunello targets each human gene with four independent sgRNAs on average and includes 1,000 non-targeting negative controls. Cas9+ P3HR-1 were transduced with Brunello at a multiplicity of infection of 0.3, to minimize lentivirus co-infection. Following transduced cell puromycin selection, cells were cultured through Day 9 post-transduction. FACSort was then performed to separate cells in which CRISPR knockout (KO) de-repressed EBV late lytic glycoprotein 350 (gp350) plasma membrane expression^5^ (Fig. 1A). sgRNA abundance in gp350+ sorted cells versus the pre-sort input population were quantitated, and STARS^38^ analysis was performed to identify screen hits, for whom multiple sgRNAs against a given gene target were significantly enriched in gp350+ cells.

At a multiple hypothesis test adjusted q<0.05 cutoff, 55 hits were identified (Fig. 1B; Table 1). Top screen hits included the genes encoding LSD1 (Lysine-specific histone demethylase 1, encoded by *KDM1A*), ZNF217 (zinc finger protein 217) and CoREST (REST Corepressor 1, encoded by *RCOR1*). Importantly, LSD1, CoREST and ZNF217 form a complex that binds DNA and that functions as a H3K4 methylation epigenetic eraser (Fig. 1B; Table 1). Cross-comparison of our Brunello and Avana sgRNA library CRISPR screen^19, 38^, which use distinct sgRNAs, further highlighted LSD1, ZNF217 and CoREST as robust CRISPR screen hits at this timepoint (Fig. 1B–C and Extended Data Fig. S1A). Notably, LSD1 scored at Day 9 but not Day 6 of the Avana screen, whereas MYC instead scored at Avana Day 6^19^, indicating potentially distinct kinetics of lytic reactivation upon their perturbation (Fig. 1C and Extended Data Fig. S1A). We validated that CRISPR MYC depletion by Brunello sgRNAs most strongly induced EBV lytic cycle immediate early BZLF1, early BMRF1 and late gp350 expression at Day 6 post-induction (Extended Data Fig. S1B–C). STAGA, mediator and cohesin subunits were enriched amongst both screen hits (Fig. 1C and Extended Data Fig. S1A).

Multiple additional CRISPR screen hits encode transcriptional repressors that can physically interact with the LSD1/ZNF217/CoREST complex, including CTBP1 (C-terminal binding protein 1), BCL6 (B-cell lymphoma 6) and PHF12 (PHD finger protein 12)^39–42^ (Fig. 1B, extended data Fig. S2A; Table 1). We validated that depletion of CTBP1, BCL6 or PHF12 induced EBV lytic gene expression and increased viral genome copy number (Extended Data Fig. S2B–S2H). Taken together, these data suggest that a complex containing LSD1, ZNF217 and CoREST is critical for maintenance of Burkitt B-cell EBV latency.

### The LSD1/ZNF217/CoREST complex restricts EBV lytic reactivation

**Figure 2.**
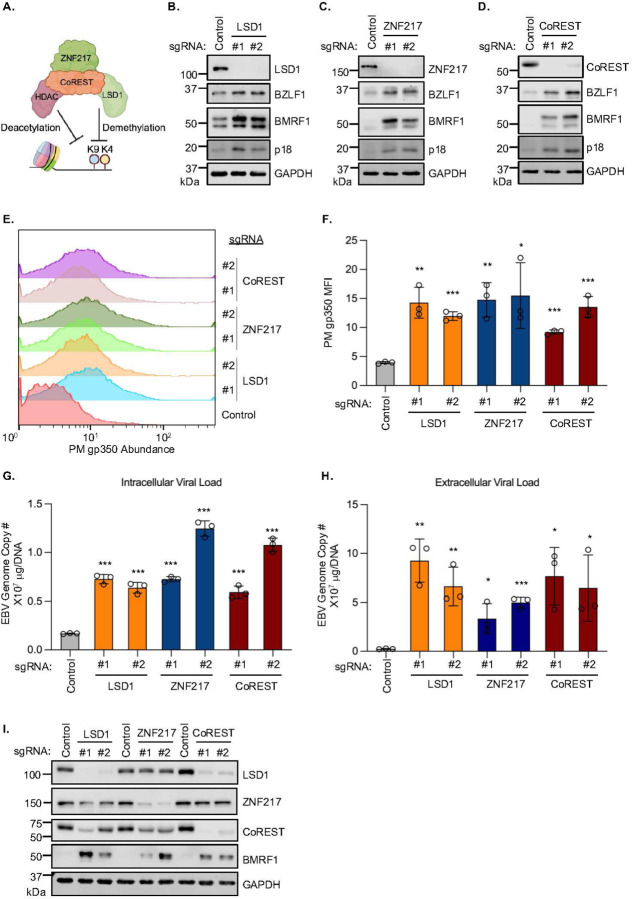
The LSD1/ZNF217/CoREST complex restricts Burkitt EBV lytic reactivation. (**A**) Schematic of the LSD1/ZNF217/CoREST complex, which can erase H3K4 methyl and/or H3K9 acetyl marks. (**B-D**) Immunoblot analysis of whole cell lysates (WCL) from Cas9+ Akata cells expressing control, LSD1 (B), ZNF217 (C), or CoREST (D) sgRNAs for immediate early lytic BZLF1, early BMRF1 and late p18 expression. (**E**) Fluorescence-activated cell sorting (FACS) analysis of PM gp350 levels on Akata cells expressing control, LSD1, ZNF217, or CoREST sgRNAs. (**F**) Mean fluorescence intensity (MFI) ± standard deviation (SD) gp350 PM values from n=3 independent replicates. (**G-H**) Intracellular (G) or extracellular (H) qPCR analysis of EBV genome copy number in Akata cells expressing control, LSD1, ZNF217, or CoREST sgRNA. Shown are mean ± SD values from n=3 replicates. P-values indicate significance of differences relative to sgRNA control cell values. (**I**) Immunoblot analysis of WCL from Akata cells expressing control, LSD1, ZNF217, or CoREST sgRNA. Blots are representative of n=3 replicates. *p<0.05, **p<0.01, ***p< 0.001.

LSD1, ZNF217 and CoREST form a complex together with class I and II histone deacetylases (HDAC) that erase H3K4 and H3K9 methylation and acetylation epigenetic marks^43–45^, each of which serve to activate or repress chromatin^46^ (Fig. 2A). We confirmed that ZNF217, CoREST, HDAC1 and 2 co-immunoprecipitated with LSD1 from P3HR-1 whole cell lysates (Extended Data Fig. S3A). Although LSD1, ZNF217 or CoREST protein abundances do not significantly change upon Burkitt cell EBV lytic reactivation^47^ (Extended Data Fig. S3B), we validated that CRISPR depletion of LSD1, ZNF217 or CoREST was sufficient reactivate EBV, as judged by immunoblot for immediate early BZLF1, early BMRF1 or late p18 expression, or by plasma membrane gp350 expression (Fig. 2B–2F, Extended Data Fig. S3C–E). Further suggesting that the LSD1/ZNF217/CoREST complex maintains EBV latency, perturbation of any of the subunits increased copies of intracellular and DNAse-resistant (encapsidated) extracellular EBV genomes, suggestive of a productive lytic replication cycle (Fig. 2G–H).

In keeping with the observation that LSD1 and CoREST stabilize one another on the protein level^48, 49^, CRISPR depletion of LSD1 markedly reduced CoREST steady state levels and vice versa. Depletion of either also reduced ZNF217 abundance, albeit to a lesser extent (Fig. 2I). CoREST depletion similarly reduced steady state LSD1 levels in EBV+ and EBV− Burkitt cells, suggesting that EBV does not contribute to their interdependence (Extended Data Fig. S3F).

### LSD1 and HDAC inhibitors additively reactivate EBV in B and epithelial cells

Potent and selective LSD1 antagonists have been developed, several of which are in clinical development. We therefore next asked whether chemical LSD1 inhibition was sufficient to reactivate EBV. The LSD1 antagonist C12 (also known as SP-2509) rapidly induced EBV lytic protein expression in multiple Burkitt cell lines, in a dose-dependent manner. C12 also induced EBV lytic protein expression in the latency III lymphoblastoid cell lines KEM III and GM15892, the gastric carcinoma AGS and in C666–1 nasopharyngeal carcinoma cells (Extended Data Fig. S4A). As little as 6 hours of exposure to C12 was sufficient to induce EBV lytic gene expression by two days post treatment (Extended Data Fig. S4B). These results suggest that LSD1 exerts pro-latency roles across EBV−transformed B and epithelial cell contexts.

**Figure 3.**
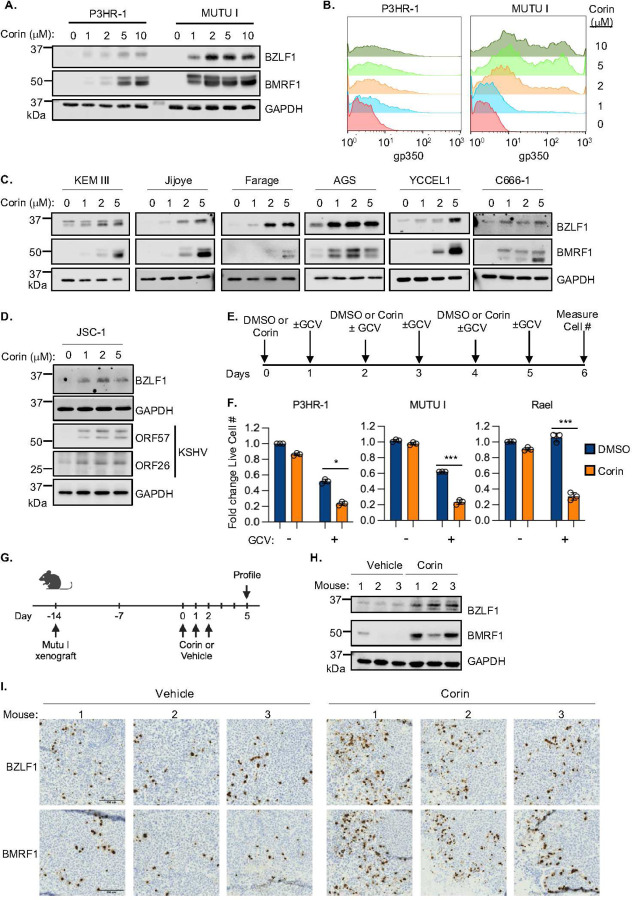
LSD1 inhibition induces EBV lytic reactivation in EBV+ cancer cells. (**A**) Immunoblot analysis of WCL from P3HR-1 or MUTU I Burkitt cells treated with the indicated Corin concentrations for two days. **(B)** FACS analysis of PM gp350 levels in Corin-treated P3HR-1 or MUTU I cells. **(C)** Immunoblot analysis of WCL from EBV+ KEM III, Jijoye, Farage, AGS, YCCEL1, C666–1 cells treated with Corin (0, 1, 2, or 5 μM) for two days. (**D**) Immunoblot analysis of WCL from EBV+/KSHV+ JSC-1 primary effusion lymphoma cells treated with Corin (0, 1, 2, or 5 μM) for two days. (**E**) Workflow of Corin and ganciclovir (GCV) treatment. Cells were seeded into fresh media on days of Corin treatment. GCV (10 μg/ml) was added twice daily where indicated. (**F**) EBV+ Burkitt B-cells were treated as described in (E), with the following Corin concentrations: P3HR-1 (0.5 μM), MUTU I (0.5 μM), Rael (0.25 μM) vs vehicle. Shown are mean ± SD live cell number relative to DMSO-treated controls on day 6 from n=3 replicates. Values of DMSO treated control cells were normalized to 1. *p<0.05, ***p< 0.001. (**G**) MUTU I murine xenograft experiment workflow. Two weeks post Mutu I Burkitt xenograft implantation, mice were treated with vehicle or Corin (30 mg/ml) as indicated. (**H**) Immunoblot analysis of WCL prepared from xenografts harvested following treatment as in (G). (**I**) Immunohistochemical analysis of BZLF1 and BMRF1 expression in xenograft tumors following treatment with vehicle vs. Corin. Scale bar = 100 μm. All blots shown are representative images of n=3 replicates.

Since HDACs associate with the LSD1/ZNF217/CoREST complex and as there is growing interest in using HDAC inhibitors for EBV lytic induction therapy^29^, we next examined effects of C12 in combination with the HDAC inhibitor sodium butyrate (NaB). C12 and NaB additively induced EBV lytic gene expression (Extended Data Fig. S4C–G). Similarly, the bifunctional LSD1 and class I HDAC inhibitor Corin induced EBV lytic protein expression in multiple Burkitt cell lines, in a dose-dependent manner (Fig. 3A–3B, Extended Data Fig. S5A–B). Corin also increased intracellular EBV genome copy number in P3HR-1 and MUTU I cells, suggestive of lytic cycle amplification (Extended Data Fig. S5C). As observed with C12, as little as six hours of Corin exposure induced EBV lytic protein expression in P3HR-1, Akata and MUTU I Burkitt cells by two days post-treatment (Extended Data Fig. S5B).

Multiple host factors important for EBV B-cell latency function by supporting MYC expression^19^. Therefore, we next tested if enforced lentiviral-driven MYC expression could bypass Corin effects on EBV reactivation. However, Corin induced EBV lytic proteins at similar levels in cells expressing control GFP or MYC (Extended Data Fig. S6A), suggesting that the LSD1 complex functions to maintain EBV latency either independently or downstream of MYC. We next tested if BZLF1 is necessary for Corin EBV reactivation, using Akata cells with CRISPR BZLF1 knockout (KO)^19^. This analysis highlighted that BZLF1 is required for Corin-induced EBV reactivation, as Corin was unable to induce BMRF1 early or p18 late protein expression in BZLF1 KO Akata cells (Extended Data Fig. S6B). These results suggest that LSD1 and HDAC restrict EBV lytic reactivation in Burkitt cells by repressing EBV immediate early gene expression.

We next tested Corin effects on EBV lytic protein expression across a range of EBV+ transformed cell states. Corin induced EBV lytic proteins in a dose-dependent manner in latency I Burkitt cells, in latency III Jijoye Burkitt cells, in the LCLs KEM III and GM15892, in Farage diffuse large B cell lymphoma cells, in AGS, YCCEL1 and SNU-719 gastric carcinoma and in C666–1 nasopharyngeal carcinoma cells (Fig. 3C and Extended Data Fig. S6C). Corin likewise induced EBV and γ-herpesvirus Kaposi’s Sarcoma Associated Herpesvirus (KSHV) ORF57 early lytic protein and ORF26 late lytic protein lytic protein expression in JSC-1 and BC-1 primary effusion lymphoma (PEL) cells (Fig. 3D and Extended Data Fig. S6D). Corin also induced KSHV lytic protein expression in BCBL1 PEL cells, which are not co-infected by EBV, as well as in iSLK219 a renal carcinoma cells^50^, where Corin induced a red fluorescence protein KSHV lytic gene reporter at similar levels as doxycycline-induced immediate early protein RTA expression (Extended Data Fig. S6D–E).

There is growing interest in ganciclovir (GCV)-based lytic reactivation therapy of EBV+ tumor cells^28–32, 51, 52^. We therefore next tested if Corin and GCV synergistically induced Burkitt cell death. While GCV did not affect viability of DMSO-treated control cells over the period of the assay, it significantly reduced live cell numbers of Corin-treated P3HR-1, MUTU I and Real Burkitt cells (Fig. 3E–F). Of note, Corin itself reduced P3HR-1 and MUTU I live cell numbers, likely in part due to effects of EBV lytic gene de-repression.

We next tested Corin effects *in vivo* in a murine xenograft Burkitt lymphoma model. After establishment of MUTU I tumors, NOD.CB17-Prkdc (NOD SCID) mice were treated with DMSO vehicle vs Corin (30mg/kg) daily for three days via intraperitoneal injection (Fig. 3G). Three days later, explanted tumors were analyzed by immunoblot, immunohistochemistry and qPCR for EBV lytic protein expression and by qPCR for EBV genome copy number. Corin derepressed BZLF1 and BMRF1 mRNA and protein expression (Fig. 3H–3I and Extended Data Fig. S6F–S6G) and significantly increased tumor intracellular EBV genome copy number (Extended Data Fig. S6H). Taken together, these data highlight LSD1 as a therapeutic target for EBV+ Burkitt lymphoma lytic induction therapy.

### LSD1/ZNF217/CoREST restricts EBV genomic H3K4 methylation

**Figure 4.**
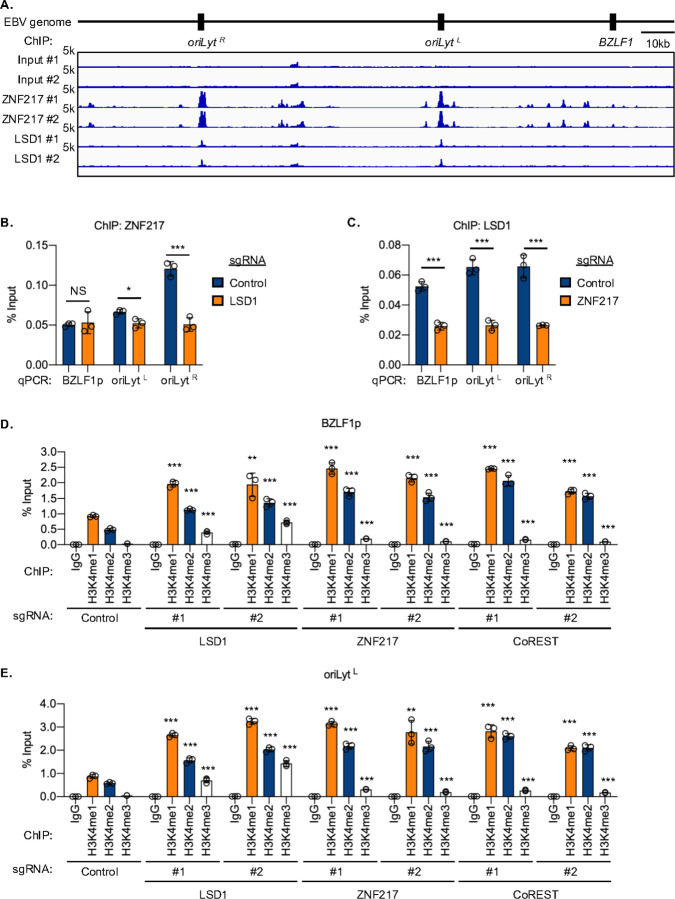
Perturbation of LSD1, ZNF217 or CoREST upregulates activating H3K4 methylation marks at the *oriLyt* enhancer and BZLF1 immediate early promoter. (**A**) Chromatin immunoprecipitation sequencing (ChIP-seq) analysis of Akata strain EBV genome-wide ZNF217 and LSD1 occupancy. Shown are input, ZNF217 and LSD1 ChIP-seq tracks from n=2 independent replicates, with track heights set to 5,000 (5K). (**B**) ChIP-qPCR analysis of ZNF217 occupancy at oriLyt versus BZLF1 promoter (BZLF1p) regions in Akata control versus LSD1 knockout (KO) cells. Mean ± SD percentages of input values from n=3 replicates are shown. (**C**) ChIP-qPCR analysis of LSD1 occupancy at *oriLyt* versus BZLF1p regions in Akata control versus ZNF217 KO, as in (B). (**D-E**) ChIP-qPCR analysis of H3K4 mono (H3K4me1), di (H3K4me2) and tri (H3K4me3) methylation levels in Akata cells expressing control sgRNA or either of two independent screen hit sgRNAs against LSD1, ZNF217 or CoRest. Shown are mean ± SD percentages of input values from n=3 replicates for *BZLF1p* (D) or *OriLyt*^*L*^ (E). Significance values in D-E refer to comparisons with control cell values. NS, not significant; *p < 0.05; **p < 0.01; ***p < 0.001.

LSD1 erases H3K4 and H3K9 mono- and di-methylation marks^43, 44, 48^. To gain mechanistic insights into how the LSD1/ZNF217/CoREST complex maintains EBV latency, we used chromatin immunoprecipitation with deep sequencing (ChIP-seq) to profile EBV genome-wide LSD1 and ZNF217 occupancy. Interestingly, ZNF217 and LSD1 highly co-occupied the *oriLyt* regions and to a lesser extent several other EBV genomic regions, including the *BZLF1* promoter (Fig. 4A). To gain further insights, we next performed ChIP-qPCR for LSD1 and for ZNF217 in control cells, in LDS1 or ZNF217 CRISPR depleted cells. LSD1 depletion more strongly decreased ZNF217 occupancy at *OriLyt* ^*R*^, whereas ZNF217 depletion similarly decreased LSD1 occupancy at the *BZLF1* promoter and at both *oriLyt* (Fig. 4B–C). Although ZNF217 supports LSD1 steady state levels, these results are consistent with a model in which ZNF217 promotes LSD1 recruitment to *oriLyt* and *BZLF1* promoter sites.

Since H3K4 methylation can activate enhancers and promoters, we next used ChIP-qPCR to test the hypothesis that the LSD1/ZNF217/CoREST complex reduces H3K4 methylation at the *BZLF1* promoter and *oriLyt* enhancer regions. Depletion of LSD1, ZNF217 or CoREST significantly increased *BZLF1* promoter and *oriLyt* H3K4me1, and to a lesser but still significant extent H3K4me2 and H3K4me3 levels (Fig. 4D–E, Extended Data Fig. S7A). By contrast, LSD1 depletion by only one of two CRISPR sgRNA very mildly increased *BZLF1* promoter and *oriLyt* region H3K9 methylation levels (Extended Data Fig. S7B). These results suggest that LSD1 H3K4 demethylase, rather than H3K9 demethylase roles, are critical for EBV latency.

Since LSD1 could play an enzymatic versus structural role in EBV genome bound complexes, we next asked if LSD1 enzymatic inhibition by C12 or Corin similarly altered H3K4 levels at the *BZLF1* promoter or *oriLyt* regions. Although C12 or Corin treatment did not affect LSD1 protein levels (Extended Data Fig. S4 and S5B), they each decreased LSD1 occupancy at the *BZLF1* promoter and at both *oriLyt* regions (Extended Data Fig. S8A). By contrast, Corin but not C12 diminished ZNF217 occupancy at these sites, possibly indicating that HDAC but not LSD1 activity is important for its ability to bind to the *BZLF1* and *oriLyt* regions. C12 and Corin each significantly upregulated H3K4 mono-, di- and tri-methylation at the *BZLF1* promoter and *oriLyt* regions (Extended Data Fig. S8B). By contrast, Corin but not C12 significantly increased *BZLF1* promoter and *oriLyt* H3K9Ac and H3K27Ac levels (Extended Data Fig. S8C). Similarly, LSD1 depletion by only one of two CRISPR sgRNA very mildly increased *BZLF1* promoter and *oriLyt* region H3K9me3 marks but decreased *BZLF1* H3K27Ac marks at these sites (Extended Data Fig. S7B and Extended Data Fig. S9A). C12 but not Corin very mildly increased H3K9me2 marks at these sites (Extended Data Fig. S9B). Taken together, these data suggest that H3K4 methylation is a major EBV genomic LSD1 epigenetic target.

### LSD1/ZNF217/CoREST restricts EBV genomic *oriLyt*/*BZLF1* DNA looping

**Figure 5.**
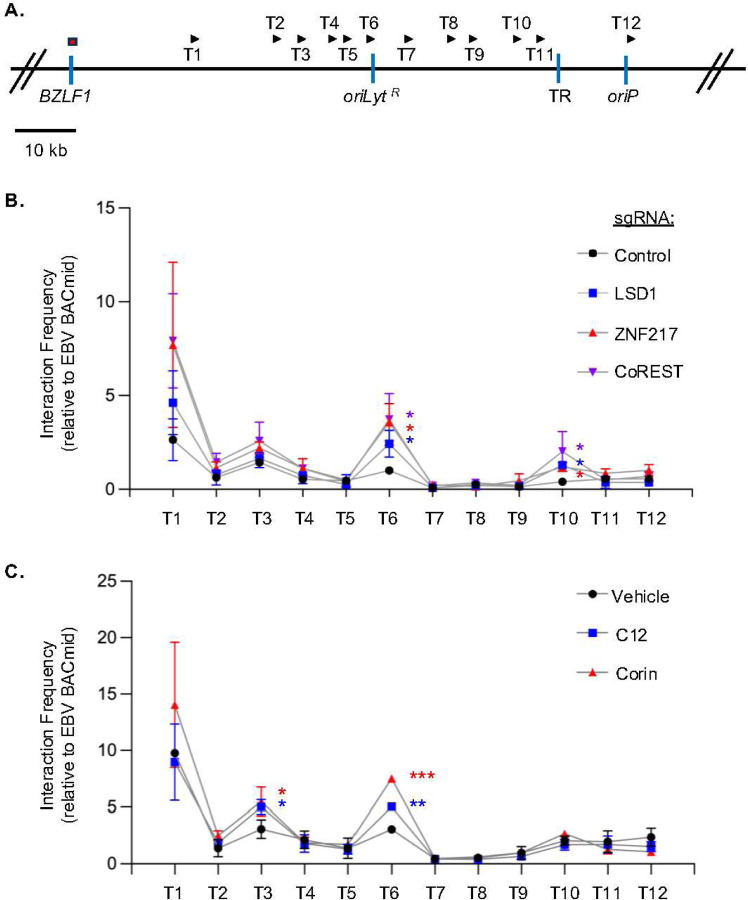
Depletion of LSD1, ZNF217 or CoREST triggers *OriLyt* and the *BZLF1* promoter region long-range DNA interaction. (**A**) Schematic diagram highlighting chromatin conformation capture (3C) assay anchor primer (red box) and 12 test (T) primers locations along with the linear EBV genome. For reference, EBV genomic terminal repeat (*TR*) and origin of plasmid replication (*OriP*) are shown. (**B**) 3C assay analysis of DNA looping between the BZLF1 anchor and 12 test primer regions. Shown are the mean ± SD 3C assay signals relative to EBV BACmid negative control from Akata cells expressing control, LSD1, ZNF217, or CoREST sgRNAs. (**C**) Mean ± SD 3C assay signals from Akata cells 24 hours post-treatment with vehicle, C12 (2.5 μM) or Corin (2.5 μM). *p < 0.05; **p < 0.01; ***p < 0.001.

Higher order EBV genomic architectural changes that juxtapose *oriLyt* enhancers with the *BZLF1* promoter are observed upon EBV B-cell reactivation^19^. We therefore asked if perturbation of LSD1, ZNF217 or CoREST altered long-range DNA looping between *oriLyt* and the *BZLF1* promoter region. Nine days following expression of control sgRNA or sgRNAs targeting the genes encoding LSD1, ZNF217 or CoREST, we performed chromatin conformation capture (3C) assays, which quantifies interaction frequencies between pairs of genomic loci^53^. As previously described^19^, a 3C assay anchor primer targeting the *BZLF1* promoter and test (T) primers tiling across the *oriLyt* ^*R*^ region, terminal repeat (TR) and nearby *oriP* region were employed (Fig. 5A). Whereas low interaction frequencies of between the *BZLF1* promoter region and either *oriLyt* ^*R*^ (T6 primer) and TR (T10 primer) were observed in control cells, their interaction frequency significantly increased upon depletion of LSD1, ZNF217 or CoREST (Fig. 5B). Similarly, 3C assay analysis identified that LSD1 inhibition by C12 or Corin treatment significantly increased interaction frequency between the *BZLF1* promoter and *oriLyt R,* suggesting on-target effects (Fig. 5C). Collectively, our data suggest that LSD1 demethylase activity in the context of a LSD1/ZNF217/CoREST co-repressor complex exerts key pro-latency roles and control higher order EBV genomic architecture.

### The H3K4 methyltransferase KMT2D supports EBV reactivation

**Figure 6.**
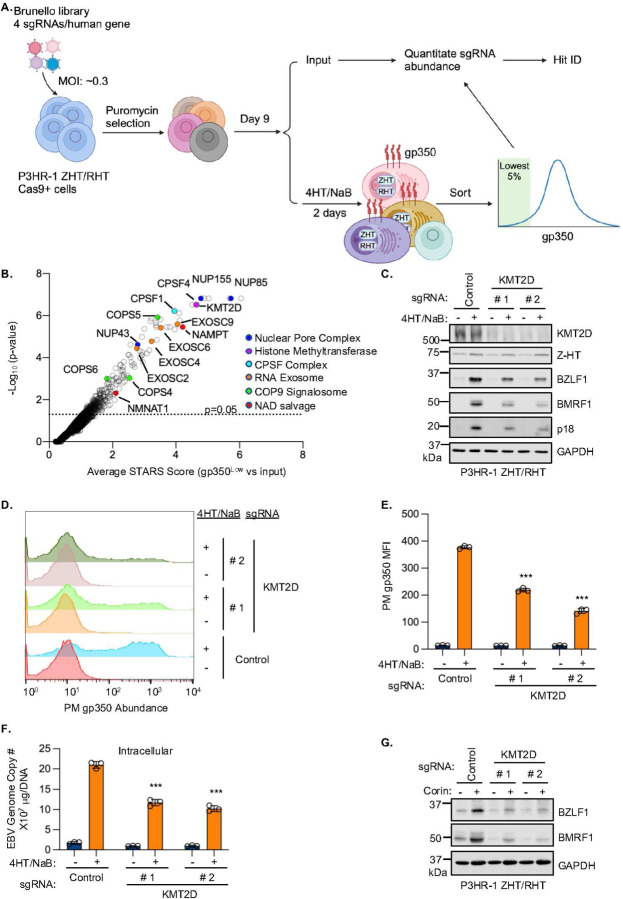
Human genome-wide CRISPR screen identifies H3K4 methyltransferase KMT2D as important for EBV reactivation. (**A**) Workflow of human genome-wide CRISPR-Cas9 screen for host factors that support EBV lytic reactivation. Cas9+ P3HR-1 with stable ZHT/RHT conditional immediate early allele expression were transduced with the Brunello sgRNA library. Transduced cells were selected and then reactivated by 4-hydroxytamoxifen (4HT, 0.4 μM) together with NaB (0.5 mM) for 48 hours. The 5% of cells with the least gp350 expression were sorted. sgRNA abundance in input vs sorted cells was quantitated to identify hits. (**B**) Volcano plot analysis of screen hits, which are highlighted by category. (**C**) Immunoblot analysis of WCL from P3HR-1 ZHT/RHT cells expressing control or KMT2D sgRNAs and mock induced or induced by 4HT (0.4 μM) and NaB (0.5 mM) for 24 hours. (**D-E**) FACS analysis of PM gp350 levels (D) and of PM gp350 MFI ± SD from n=3 replicates (E) in P3HR-1 ZHT/RHT cells with control vs KMT2D sgRNAs and mock induced or induced for lytic replication by 4HT and NaB for 24 hours. (**F**) qPCR of intracellular EBV genome copy number in P3HR-1 ZHT/RHT cells expressing control or KMT2D sgRNA that were treated with or without 4HT (0.4 μM) and NaB (0.5 mM) for 24 hours. (**G**) Immunoblot analysis of WCL from P3HR-1 ZHT/RHT cells expressing control or KMT2D sgRNA that were treated with or without Corin (2.5 μM) for 24 hours. All blots shown are representative images of n = 3 replicates. Bar graphs are presented as mean ± SD from three replicates. ***p < 0.001.

To gain further insights into the EBV lytic switch, we next performed a human genome-wide CRISPR-Cas9 screen for host factors that instead support EBV reactivation. We used the Brunello sgRNA library to transduce Cas9+ P3HR-1 cells, which had stable expression of a conditional system that triggers EBV reactivation, comprised of the EBV immediate early proteins ZTA and RTA fused to a 4-hydroxy tamoxifen (4HT)-dependent mutant estrogen receptor binding domain (termed ZHT and RHT, respectively)^54^. The addition of 4HT causes ZHT/RHT nuclear translocation, triggering EBV lytic replication, which was further enhanced by co-treatment with HDAC inhibition by NaB. At day 9 post-Brunello sgRNA library transduction, lytic reactivation was induced by 4HT and NaB for 48 hours. Cells with the lowest 5% of gp350 expression were then sorted (Fig. 6A). PCR-amplified sgRNA abundances were quantified by next-generation DNA sequencing of unsorted input library versus FACSorted cells. Using the STARS algorithm, 53 hits were identified at a multiple hypothesis test adjusted q<0.05 cutoff. Interestingly, the gene encoding the H3K4 lysine methyltransferase 2D (KMT2D, also known as MLL2 and MLL4), which writes H3K4 mono-methyl marks and which directly counteracts LSD1^55^, was a top hit. Additional hits included genes encoding and multiple components of the nuclear pore complex, the cleavage and polyadenylation specificity factor (CPSF), exosome complex, COP9 signalosome and nicotinamide adenine dinucleotide (NAD) salvage pathway (Fig. 6B; Table 2).

Given the above findings that H3K4 methylation is as major determinant of EBV latency, we used two Brunello sgRNAs to validate and to further characterize effects of KMT2D depletion on EBV lytic gene induction. Both guides efficiently depleted KMT2D without appreciably altering ZHT levels (Fig. 6C). By contrast, both guides reduced levels of BZLF1, BMRF1 and p18 in 4HT/NaB-treated cells. Since the ZHT allele functions in part by inducing endogenous BZLF1 expression, these results are consistent with a KMT2D role in regulation of the *BZLF1* promoter, with downstream effects on early and late gene induction. Consistent with our immunoblot results, KMT2D KO impaired EBV lytic cycle upregulation of plasma membrane gp350 expression (Fig. 6D–6E) and intracellular EBV genome copy number (Fig. 6F) in 4HT/NaB treated P3HR-1 ZHT/RHT cells. Similar results were observed in Akata cells triggered for lytic induction by immunoglobulin cross-linking, in which KMT2D depletion reduced immediate early, early and late protein expression and reduced intracellular EBV genome copy number (Extended Data Fig. S10A–D).

**Figure 7.**
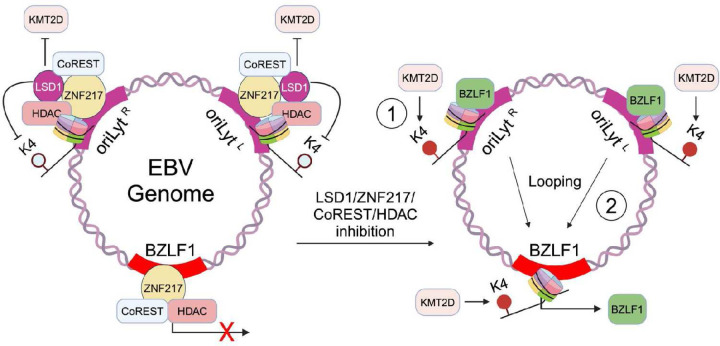
Schematic model. In latency, LSD1/ZNF217/CoREST/HDAC complexes co-occupy EBV oriLyt enhancer regions, where LSD1 and HDAC erase activating H3K4 methylation and H3K4 acetylation marks, respectively. Perturbation of LSD1 and HDAC activity enables KMT2D to deposit activating H3K4 epigenetic marks at both *oriLyt* enhancers and supports their looping to the immediate early BZLF1 promoter. Newly synthesized BZLF1 drives the lytic cycle by binding to early gene promoters and also to *oriLyt* enhancers to increase their strength, which further upregulates BZLF1 in a positive feedback loop.

Finally, we asked if KMT2D depletion could counteract Corin effects on EBV reactivation. Intriguingly, immunoblot analysis revealed that KMT2D depletion strongly impaired immediate early BZLF1 and early BMRF1 induction by Corin in P3HR-1 (Fig. 6G). Similar results were observed in Akata cells, where KMT2D depletion reduced BZLF1 and BMRF1 expression following immunoglobulin cross-linking (Extended Data Fig. S10E). Collectively, our data support a model in which KMT2D activity supports EBV reactivation whereas LSD1 activity supports EBV latency by controlling *oriLyt* and *BZLF1* promoter region H3K4 methylation levels and higher order EBV architecture (Fig. 7).

## Discussion

Here, we used complementary CRISPR-Cas9 screen approaches to characterize key host factors important for EBV latency versus reactivation in Burkitt B-cells. These highlighted opposing roles of the H3K4 methylation eraser LSD1/ZNF217/CoREST complex versus the writer KMT2D. LSD1 played a key role in maintenance of EBV latency across a range of B-cell and epithelial cell contexts, including in gastric and nasopharyngeal carcinoma cells. LSD1 and associated HDACs exerted additive roles in maintenance of EBV latency. LSD1/ZNF217/CoREST regulated higher order EBV genomic configuration to prevent looping of *oriLyt* enhancers to the *BZLF1* promoter region, which occurs with EBV reactivation^19^. KTM2D instead supported EBV lytic gene expression, likely at the level of H3K4 methylation.

While DNA methylation is important for EBV latency^23^, we and others previously identified that the histone loaders CAF1, HIRA and ATRX are also host factors that repress the EBV lytic cycle^19, 35, 56^. We also recently identified that linker histone H1, which is important for DNA compaction, is also critical for EBV latency^36^. Multiple studies have identified repressive histone marks important for EBV latency, including H3K9 and H3K27 trimethylation^35, 57–60^. Nonetheless, our present study suggests that H3K4 methylation at *oriLyt* and potentially also at the *BZLF1* promoter can overcome these repressive mechanisms, because LSD1, CoREST and ZNF217 were each found to be important for the maintenance of EBV latency.

A key question remains how LSD1, ZNF217 and presumably also CoREST is recruited to *oriLyt* in EBV latency, as observed in our ChIP-seq analyses. LSD1 is not known to have sequence specific DNA binding, and we therefore hypothesize that ZNF217 may bind to specific EBV genomic sites and recruit LSD1 and CoREST to these sites. Further experiments will be required to define whether the ZNF217 DNA binding domain itself is required for EBV latency. Since we also found that MYC occupancy of *oriLyt* E-box sites are also critical for latency^19^, it is also plausible MYC may participate in recruitment of LSD1/ZNF217/CoREST to *oriLyt* sites.

Our results suggest that changes in LSD1, ZNF217 and/or CoREST levels or activity may be a physiological mechanism by which the EBV lytic switch is regulated. Notably, LSD1 is implicated in plasma cell differentiation, which is a trigger for EBV reactivation^61^. Unfortunately, EBV reactivation in primary B cell models, including plasma cells, are not presently available and EBV does not infect mice. Humanized mouse models can be infected, but do not exhibit significant plasma cell differentiation. Nonetheless, LSD1 is implicated together with the repressor Blimp-1 in murine plasma cell differentiation^62^, as well as in murine plasmablast differentiation^63^. Furthermore, LSD1 is important for the formation of murine germinal center B-cells^41^, from which most memory B-cells arise. We therefore speculate that EBV evolved a mechanism to utilize LSD1 activity in order to maintain EBV latency, but that LSD1 activity and/or association with key EBV genomic sites are perturbed as infected memory cells undergo plasma cell differentiation and reactivate.

Although KMT2D can have partially redundant phenotypes with KMT2C, one of six H3K4 methyltransferases encoded by mammalian genomes, KMT2D activity is critical in many contexts^55^. Our CRISPR analyses identified KMT2D as the key H3K4 methyltransferase that supports Burkitt B-cell EBV lytic reactivation. While not previously studied in the context of EBV infection, KMT2D is a well characterized regulator of B-cell differentiation and lymphomagenesis. KMT2D conditional knockout in pre-B-cells increases B-cell proliferation, inhibits B-cell differentiation and promotes B-cell lymphomagenesis in mice^64, 65^. Thus, an important objective will be to determine whether KMT2D levels and/or activity are altered in human EBV−infected memory B-cells upon their plasma cell differentiation as models become available to study this transition associated with EBV reactivation.

KMT2D catalyzes H3K4mono- and dimethylation, predominantly at enhancers to drive their activation^55, 66^. LSD1 instead erases these marks. We therefore speculate that *oriLyt* enhancer H3K4 mono- and di-methylation levels are a critical determinant of EBV reactivation, though it remains to be determined how H3K4me1 controls DNA looping between *oriLyt* and the *BZLF1* promoter. One possibility is that changes in H3K4 methylation alter local transcription, which in turn reduce DNA methylation. Since the DNA loop anchoring protein CTCF (CCCTC-Binding Factor) binds unmethylated DNA, such altered EBV genomic methylation could change CTCF binding. For instance, transcription driven changes in DNA methylation control CTCF and cohesin-mediated enhancer/promoter DNA looping at the protocadherin alpha locus^67^. Notably, the H3K4 writer KMT2A is recruited to the BZLF1 promoter and promotes its expression in EBV+ gastric carcinoma cells^68^. Thus, it is plausible that KMT2D and KMT2A play analogous roles in EBV−infected B versus epithelial cell reactivation.

LSD1 and CoREST are major regulators of the alpha-herpesvirus herpes simplex virus 1 (HSV-1) gene expression^69–71^. Our results suggest that EBV and HSV-1 subvert LSD1 in distinct manners to regulate lytic gene expression. LSD1 is recruited to HSV-1 immediate early promoter regions in a large epigenetic complex that includes HCF-1 (Host Cell Factor-1)^69–71^. In this context, LSD1 demethylase activity, together with that of JMJD2 family members, erase repressive H3K9 methylation marks to promote HSV-1 immediate early gene expression. By contrast, we found that LSD1 erases EBV genomic H3K4 methylation marks to maintain EBV latency and to prevent long-range interactions between *oriLyt* enhancer and *BZLF1* promoter regions. LSD1 inhibition triggered a full EBV lytic cycle, suggesting that LSD1 does not promote EBV early or late gene expression. While LSD1 activity is not required for HSV lytic replication phase I, where a synchronous lytic transcript wave is observed 20 hours post-stimulus, LSD1 activity is instead critical for full reactivation and viral lytic DNA replication^71–74^. Interestingly, LSD1 inhibition also blocks lytic gene expression of the beta-herpesvirus cytomegalovirus and of adenovirus type 5^72^. These results indicate that gamma-herpesviruses evolved to bypass LSD1 roles following immediate early gene de-repression.

EBV and HSV-1 also distinctly utilize CoREST to regulate lytic gene expression. Whereas CoREST repressed all classes of EBV lytic genes, it instead supports HSV-1 immediate early gene expression^75–77^. In fact, HSV immediate early ICP0 shares a domain with CoREST and targets CoREST for degradation. This ICP0 activity re-localizes HDAC1/2 to the cytoplasm, promoting histone acetylation, early and late gene expression^75, 76, 78^. While the neuronal gene repressor REST is also implicated in HSV lytic gene regulation, our CRISPR analyses did not reveal a REST role in regulation of EBV lytic genes, even though it is expressed in EBV+ B-cells^79^. Thus, our results suggest that EBV has coopted a distinct LSD1/ZNF217/CoREST complex to repress the lytic switch.

There is considerable interest in translational approaches to trigger EBV lytic reactivation to sensitize infected tumors to the antiviral ganciclovir^29^. For instance, the HDAC inhibitor arginine butyrate together with ganciclovir exhibited activity in a phase I/2 clinical trial. 10 of 15 patients with refractory EBV+ lymphoid malignancies showed antitumor responses in this study, but most patients did not achieve a complete remission, suggesting the need for further optimization^80^. Our results raise the possibility that combinatorial LSD1 and HDAC inhibition may improve clinical responses.

In summary, human genome-wide CRISPR/Cas9 screens highlighted key roles for LSD1, ZNF217, and CoREST in maintenance of EBV latency in B and epithelial cell contexts. CRISPR or chemical LSD1 inhibition, in particular together with HDAC inhibition, drove EBV immediate early, early and late protein expression and EBV genome amplification. Combined LSD1/HDAC inhibition de-repressed EBV lytic gene expression, both *in vitro* and *in vivo*, and sensitized EBV+ Burkitt cells to ganciclovir toxicity, suggesting novel EBV lytic induction therapeutic approaches.

## Materials and Methods

### Cell culture and chemicals

All cell lines, including EBV−positive Burkitt lymphoma cells (P3HR-1, P3HR-1 ZHT/RHT, Akata, MUTU I, KEM I, EB3, Rael, Jijoye), EBV−positive lymphoblastoid cell lines (GM15892, KEM III), EBV−positive gastric carcinoma cells (AGS, YCCEL1, SNU719), EBV−positive nasopharyngeal carcinoma cells (C666–1), EBV−positive diffuse large B-cell lymphoma (Farage), primary effusion lymphoma cells (EBV+/KSHV+ BC-1 and JSC-1; KSHV+ BCBL-1), were cultured in Roswell Park Memorial Institute (RPMI) 1640 medium supplemented with 10% fetal bovine serum (FBS). The P3HR-1 ZHT/RHT cells carry a conditional BZLF1 allele (ZHT) and BRLF1 allele (RHT) fused with a 4-Hydroxytamoxifen (4HT) responsive estrogen receptor at carboxyl terminus. Without 4HT, ZHT/RHT retains in the cytosol, while addition of 4HT stabilizes and translocates ZHT/RHT to nuclear. Akata cells stably expressing BZLF1 sgRNA, GFP cDNA, or HA-MYC cDNA were constructed previously and maintained in our laboratory^19^. 293T and KSHV+ iSLK219 cells were cultured in Dulbecco’s Modified Eagle’s Medium (DMEM) with 10% FBS. The iSLK219 stably express a doxycycline-inducible RTA and an RFP-GFP-Puro construct, where GFP is controlled by EF1α promoter and RFP is controlled by lytic PAN RNA promoter. Addition of doxycycline induces expression of RTA and then triggers the KSHV lytic reactivation. In this study, all cell lines, except EB3, 293T, and iSLK219, are stably express *Streptococcus pyogenes* Cas9 engineered via lentiviral transduction and blasticidin selection. Cell cultures were maintained in a humidified chamber at 37°C with 5% carbon dioxide.

Puromycin was used at 3 μg/ml for selection of transduced cells. LSD1 inhibitor C12 (also known as SP-2509, 0–10 μM), LSD1 and HDAC dual inhibitor Corin (0–10 μM), HDAC inhibitor sodium butyrate (NaB, 0.5 mM), 4HT (0.4 μM), doxycycline (Dox, 0.5 μg/ml), ganciclovir (GCV, 10 μg/ml), and anti-human IgG (10 μg/ml), were all purchased commercially and are listed in Extended Data Table S1.

### Genome-wide CRISPR-Cas9 screen

One hundred and fifty million Cas9+ P3HR-1 cells were spin-inoculated for 2 hours at 300 g in the presence of 4 μg/ml polybrene in 12-well tissue culture plates, with Brunello sgRNA library^37^ at multiplicity of infection (MOI) of ~0.3. Plates were incubated at 37°C for 6 hours after spin-inoculation and then were cultured in fresh growth medium at 0.3 million/ml density. Puromycin (3 μg/ml) was added at 48 hours post transduction and cells were passaged every three days. At day 9 post transduction, 40 million cells were harvested as input control for genomic DNA isolation using QIAGEN Blood and Cell Culture DNA Maxi Kit. One hundred and sixty million cells were stained with Cy5 conjugated anti-gp350 antibody (clone 72A1) and were subjected to FACS sorting at Human Immunology Center Flow Core in Brigham and Women’s Hospital, Boston, MA. One hundred and sixty million cells were induced with 4HT (0.4 μM) and NaB (0.5 mM) for two days and then were subjected to staining with anti-gp350-Cy5 antibody and FACS sorting. Genomic DNA of sorted cells were isolated with QIAGEN DNeasy Blood & Tissue Kit. All DNA were sent for PCR amplification and sequencing to quantify the sgRNA abundance. Statistically significant screen hits were identified following the STARS algorithm analysis^38^.

### Individual CRISPR-Cas9 knockout

Individual single guide RNA (sgRNA) directed CRISPR-Cas9 gene knockout was performed as previously described^81^. Briefly, the sgRNA from Brunello library was cloned into pLentiGuide-puro (a gift from Feng Zhang, Addgene plasmid #52963) and confirmed by sequencing. Cells were transduced with lentivirus that was produced by transfecting 293T cells with pCMV-VSV-G (a gift from Bob Weinberg, Addgene plasmid #8454), psPAX2 (a gift from Didier Trono, Addgene plasmid #12260), and the sgRNA expression vector using TransIT-LT1 transfection reagent. Transduced cells expressing a non-target control sgRNA or target gene sgRNA were selected with puromycin (3 μg/ml) for 3 days. The on-target effects of CRISPR were verified by immunoblotting. All sgRNA used in this study are listed in Extended Data Table S2.

### Flow cytometry analysis

Cells were pelleted and washed once with FACS buffer (2% FBS v/v in PBS), and then were stained with anti-gp350-Cy5 (1 μg/ml) for 30 minutes at room temperature in the dark. The labeled cells were then pelleted, washed twice, and re-suspended in FACS buffer within flow cytometry-compatible tubes and processed immediately with BD FACSCalibur instrument. All FACS data was analyzed using FlowJo X software.

### Immunoblotting and Immunofluorescence

Immunoblotting was performed following previously described procedures^23^. Cells were lysed in 1x Laemmli Sample Buffer and sonicated briefly. The whole cell lysates were separated via SDS-PAGE electrophoresis and subsequently transferred onto nitrocellulose membranes. The membranes were then blocked with 5% nonfat milk in TBST (0.1% Tween 20 in TBS) for one hour and incubated overnight at 4 °C with primary antibodies. The following day, the blots were washed three times with TBST and were incubated with secondary antibodies for one hour at room temperature. After three additional washes with TBST, the blots were developed using ECL chemiluminescence substrate and imaged with the LI-COR Fc platform. All antibodies used in this study are listed in Extended Data Table S1.

The iSLK219 cells seeded in 12-well plates were treated with Dox (0.5 μg/ml) or Corin (5 μM) for 24 hours and then cultured in fresh 10% FBS DMEM without chemicals. Two days later, cells were visualized and recorded using the EVOS M7000 cell imaging system (Invitrogen).

### EBV genome copy number

DNA in cells and supernatant were extracted using the QIAGEN DNeasy Blood & Tissue Kit as previously described^19^. Intracellular and extracellular EBV genome copy numbers were quantified via qPCR. Ten-fold serial dilutions of the pHAGE-BALF5 plasmid were used to establish the standard curve. The extracted DNA was then diluted to a concentration of 10 ng/ml and subjected to qPCR with primers target EBV BALF5 (primers listed in Extended Data Table S2). The viral DNA copy numbers were calculated by plotting the sample cycle threshold (Ct) values into the regression equation derived from the standard curve.

### Co-immunoprecipitation

A total of 150 million cells were harvested and lysed in a cold lysis buffer (1% v/v NP40, 150 mM Tris, and 300 mM NaCl) supplemented with 1X cOmplete EDTA-free protease inhibitor, 1mM Na_3_VO_4_, and 1mM NaF for 1 hour at 4 °C with rotation. The lysed cells were then pelleted, and 1% supernatant was taken as input. The remaining lysates were incubated with anti-LSD1 or normal IgG with 20 μl protein A/G magnetic beads at 4 °C overnight with gentle rotation. Next day, magnetic beads were washed five times with lysis buffer and then eluted using 1X SDS loading buffer, incubated for 10 minutes at 95 °C along with input. Immunoprecipitated samples and input were subjected to immunoblotting as described above.

### Mouse xenograft experiments

Mouse xenograft experiments were performed in accordance with Institutional Animal Care & Use Committee (IACUC #2022–0002) protocol regulations at Weill Cornell Medical Center. NOD.CB17-Prkdc (NOD scid) mice were purchased from Jackson Laboratories. Six to eight-week-old male and female mice (3 males/3 females) were injected subcutaneously with 1x10^7^ MUTU I cells suspended in PBS with Matrigel in each flank. Once the tumors reach approximately 100 mm^3^, mice were treated with vehicle (5% DMSO) or Corin (30 mg/kg) daily for three days via intraperitoneal injections. Mice were humanely sacrificed three days after the last treatment. Tumors were harvested for RNA, DNA, and protein extraction, and sectioned for immunohistochemistry.

### Immunohistochemistry

Tumor samples were fixed in 10% neutral buffered formalin and were embedded into paraffin wax. 7 mm sections of tumors were stained with the primary antibodies BZLF1 (Santa Cruz, sc-53904) or BMRF1 (GeneTex, GTX30757), followed by staining with HRP-conjugated goat anti-mouse antibody for 1h at 37 °C. Tumor sections were further stained with Hematoxylin and eosin (H&E).

### Quantitative real time (qRT)-PCR

Total RNA was extracted from tumor samples using the RNeasy Mini Kit (QIAGEN) with DNase treatment. Reverse transcription was performed using iScript Reverse Transcription Supermix (BIO-RAD). The synthesized cDNA was subjected to qRT-PCR using PowerUp SYBR Green Master Mix (Applied Biosystems) on an CFX96 Touch Real-Time PCR Detection System (BIO-RAD). All data were normalized to signal of 18S rRNA and relative gene expression was calculated by 2^-ΔΔCt^ method. All used primer sequences were listed in the Extended Data Table S2.

### Chromatin immunoprecipitation (ChIP) and ChIP sequencing (ChIP-seq) assays

Akata cells were crosslinked with a 1% formaldehyde solution in growth medium and then quenched with 2.5M glycine in distilled water. The cells were washed with ice-cold PBS and lysed in 1% SDS lysis buffer (50 mM Tris, 10 mM EDTA, and 1% SDS) supplemented with 1X cOmplete EDTA-free protease inhibitor. Chromatin was sheared to 100–300 bp with Bioruptor Pico sonication, followed by centrifugation at 15,000 x g for 10 minutes at 4 °C. The resulting supernatant was diluted at a 1:10 ratio in ChIP dilution buffer (1.2 mM EDTA, 16.7 mM Tris, 167 mM NaCl, 0.01% SDS, and 1.1% Triton X-100) supplemented with 1X cOmplete EDTA-free protease inhibitor. 1% of the sheared chromatin was saved as Input and stored at −80 °C until use. The diluted chromatin was rotated overnight at 4 °C with antibody and 20 μl protein A/G magnetic beads. Next day, the immunocomplexes were subsequently washed twice with low-salt buffer (150 mM NaCl, 2 mM EDTA, 20 mM Tris-HCl, 0.1% SDS, 1% Triton X-100) and high-salt buffer (500 mM NaCl, 2 mM EDTA, 20 mM Tris-HCl, 0.1% SDS, 1% Triton X-100), and once with LiCl buffer (0.25 M LiCl, 1% NP-40, 1% sodium deoxycholate, 1 mM EDTA, 10 mM Tris) and TE buffer (1 mM EDTA, 10 mM Tris). After eluting in Elution buffer (100 mM NaHCO3, 1% SDS), DNA was reverse cross-linked for 2 hours at 65 °C. Immunoprecipitated DNA was purified with QIAquick PCR purification kit, followed by qPCR with primers specific to EBV promoters. For ZNF217 and LSD1 ChIP-seq, Akata cells were fixed and subjected to ChIP as stated above. Each antibody ChIP was performed in biological duplicate. After elution, immunoprecipitated DNA were subjected to library preparation using NEBNext Ultra II DNA Library Prep Kit and were sequenced on an Illumina NovaSeq 6000 using PE150 Sequencing Strategy by Novogene Corporation. All antibodies and primers used for ChIP are listed in Extended Data Table S2.

### ChIP-seq data analysis

ChIP-seq reads were first aligned to the Akata EBV genome using Bowtie2 v2.5.1^82^ with the parameters -*k 1"*. PCR duplicated reads were marked and removed using *Picard* (http://broadinstitute.github.io/picard/) and *Samtools* ^83^. The EBV tracks were normalized to total mapping reads and generated using *bedtools* (https://github.com/arq5x/bedtools2) v2.30.0 with the parameters “genomecov -bga -split -scale” together with *bedGraghToBigWig* (http://hgdownload.cse.ucsc.edu/admin/exe/linux.x86_64/bedGraphToBigWig). The tracks were visualized using Integrative Genomics Viewer (IGV).

### Chromosome conformation capture (3C) assay

The 3C assay was performed as previously described^19^. Ten million of Akata cells were palleted and resuspended in PBS containing 10% FBS and crosslinked with 2% formaldehyde for 10 minutes at room temperature with rotation. Cells were quenched with ice-cold 0.125M glycine and washed with ice-cold PBS. Cells were then pelleted and lysed with 5ml of ice-cold lysis buffer (10mM Tris-HCl pH:8.0, 10mM NaCl, 0.2% NP-40) supplemented with 1X cOmplete EDTA-free protease inhibitor for 10 minutes on ice. As the ligation control, 10 μg EBV wild type BACmid DNA was included and processed same as cell samples in the following steps. Nuclei were then pelleted and resuspended in 0.5 ml 1.2x Csp6I Fastdigest restriction buffer. SDS was added at a final concentration of 0.3%, and nuclei were incubated for 1 hour at 37 °C with shaking at 900 rpm. Subsequently, 2% final concentration of Triton X-100 was added to the solution, followed by one hour incubation at 37 °C with shaking at 900 rpm. After adding 400 U Csp6I digestion enzyme, the samples were incubated at 37 °C overnight with shaking at 900 rpm. To assess digestion efficiency, 10 μl of samples were collected before and after the Csp6I reaction. The reaction was halted by the addition of a final concentration of 1.6% SDS and incubated at 65 °C for 30 minutes while shaking at 900 rpm. Samples were then diluted 10-fold with 1.15X T4 DNA ligase reaction buffer and 1% final concentration of Triton X-100 was added. The samples were then incubated for 1 hour at 37 °C while shaking at 900 rpm. Subsequently, 100 U T4 DNA ligase was added, and samples were incubated at 16 °C for 4 hours, followed by a 30-minute incubation at room temperature. After adding 300 μg proteinase K, samples were incubated at 65 °C overnight. Next day, samples were treated with 300 μg of RNase A for 1 h at 37 °C. Then, DNA was purified with phenol-chloroform and ethanol precipitation. The purified DNA was subjected by qPCR with primers across EBV *BZLF1* and *oriLyt* regions (Extended Data Table S2). The ΔCt method was utilized to analyze 3C-qPCR data, where Ct value of each primer pair from each sample were normalized to Ct value of EBV BACmid DNA control random ligation product.

### Quantification and statistical analysis

Statistical significance of CRISPR-Cas9 screen results was analyzed using STARS algorithm v1.3 to compare the sgRNA abundances in FACS sorted cells versus input library. All FACS, immunofluorescent images, and blots show representative images from n =3 replicates. All bar and line graphs are shown as mean ± standard deviation (SD) of n = 3 replicates. Statistical significance between indicated groups was determined using the student’s t-test with GraphPad Prism 8 software, where NS denotes not significant (*p* > 0.05), * *p* < 0.05, ** *p* < 0.01, and *** *p* < 0.001.

## Supplementary Material

Supplement 1 Table 1. CRISPR-Cas9 screen hits.
Table 2. CRISPR-Cas9 4HT+NaB screen hits.
