# Supplementary material for: The Histone Demethylase LSD1/ZNF217/CoREST Complex is a Major Restriction Factor of Epstein-Barr Virus Lytic Reactivation
Source: Res Sq. 2025 Jan 13:rs.3.rs-5649616. Preprint. [Version 1] doi: 10.21203/rs.3.rs-5649616/v1 (PMC11774438; doi:10.21203/rs.3.rs-5649616/v1)
Supplement: Supplement 1 — Table 1. CRISPR-Cas9 screen hits. Table 2. CRISPR-Cas9 4HT+NaB screen hits. [file NIHPPRS5649616v1-supplement-1.pdf]

Extended Data Figure Legends

Extended Data Figure S1. MYC knockout rapidly triggers EBV lytic reactivation.
(**A**) Cross-comparison of Avana sgRNA library Day 6 versus Brunello library Day 9 CRISPR screens for host factors that repress EBV reactivation. (**B**) Immunoblot analysis of WCL from P3HR-1 (left) or Akata (right) cells that expressed control vs Brunello MYC targeting sgRNAs at the indicated days post transduction. All blots shown are representative of n = 3 replicates. (**C**) Mean ± SD PM gp350 levels from n=3 replicates of P3HR-1 (left) or Akata (right) cells expressing control vs MYC sgRNAs as in (B). NS: not significant, *** $p < 0.001$.

Extended Data Figure S2. LSD1/ZNF217/CoREST complex co-repressors restrict Burkitt B-cell EBV reactivation.
(**A**) STRING interaction network analysis[84] of LSD1/ZNF217/CoREST complex and its associated factors. Shown are edges, which depict protein-protein associations, and confidence scores, which depict the estimated likelihood, based on supporting evidence, that the predicted interaction is biologically meaningful, specific and reproducible. (**B**) Immunoblot analysis of WCL from Akata cells expressing control or CTBP1 targeting sgRNA. (**C**) FACS analysis of PM gp350 abundance in Akata cells expressing control or independent Brunello library CTBP1 targeting sgRNAs. (**D**) Immunoblot analysis of WCL from Akata cells expressing control or BCL6 targeting sgRNA. (**E**) FACS analysis of PM gp350 levels in Akata cells expressing control or BCL6 targeting sgRNA. (**F**) Immunoblot analysis of WCL from Akata cells expressing control or independent Brunello PHF12 sgRNAs. (**G**) FACS analysis of PM gp350 levels in Akata cells expressing control or independent Brunello PHF12 targeting sgRNAs. (**H**) Mean ± SD intracellular EBV genome copy number from n=3 replicates of Akata cells expressing control, CTBP1, BCL6, or PHF12 sgRNAs. Blots shown are representative of n=3 replicates. ** $p < 0.01$, *** $p < 0.001$.

Extended Data Figure S3. LSD1, ZNF217 and CoREST maintain Burkitt B-cell EBV latency.
(**A**) WCL of P3HR-1 cells were subjected to immunoprecipitation using control IgG or anti-LSD1 antibody, followed by immunoblot analysis with 1% Input for the indicated proteins. (**B**) Left, LSD1, ZNF217 and CoREST relative protein abundances in P3HR-1 ZHT/RHT cells uninduced or induced for lytic replication by 4-HT for the indicated times (right) or in EBV-negative (EBV-) versus EBV-positive (EBV+) Akata mock induced or induced for lytic replication by anti-IgG crosslinking for 48 hours, using data from Ersing et al.[47]. (**C-E**) FACS analysis of PM gp350 abundances in P3HR-1 cells expressing control, LSD1 (C), ZNF217 (D) or CoREST (E) targeting sgRNAs. (**F**) Immunoblot analysis of WCL from EBV+ or EBV- Akata cells expressing control or CoREST sgRNA. Blots are representative of n=3 independent replicates.

Extended Data Figure S4. LSD1 inhibition by the small molecule antagonist C12 induces EBV reactivation in B and epithelial cells.
(**A**) Immunoblot analysis of WCL from latency I EB3 or Rael Burkitt cells, latency III GM15892 LCLs or KEM III LCLs, EBV+ AGS gastric carcinoma and C666-1

nasopharyngeal carcinoma cells treated with C12 (0,1,2 or 5 μM) for 48 hours. (**B**) Immunoblot analysis of WCL from MUTU I that were treated with the indicated concentration of C12 for 6 or 24 hours and then grown in C12-free RPMI for the remainder of 48 hours. (**C**) Immunoblot analysis of WCL from KEM I cells treated with C12 (0, 1, 2, 5, or, 10 μM) alone or in combination with NaB (0.5 mM) for 48 hours. (**D-E**) Immunoblot analysis of WCL (D) and FACS analysis of PM gp350 (E) from P3HR-1 cells treated with C12 (0, 1, 2, 5, or, 10 μM) alone or in combination with NaB (0.5 mM) for 48 hours. (**F-G**) Immunoblot analysis of WCL (F) and FACS analysis of PM gp350 levels (G) from MUTU I cells treated with C12 (0, 1, 2, 5, or, 10 μM) alone or in combination with NaB (0.5 mM) for 48 hours. Shown in the top right of the panels in (E) and (G) are %gp350+ cells. Blots are representative of n=3 independent replicates.

Extended Data Figure S5. Corin induces Burkitt B-cell EBV lytic reactivation.
(**A**) Schematic diagram depicting bifunctional small molecule antagonist Corin inhibition of LSD1/ZNF217/CoREST/HDAC complex LSD1 and HDAC activity. (**B**) Immunoblot analysis of WCL from P3HR-1, Akata or MUTU I cells that were treated with the indicated concentration of Corin for 6 hours and then growth Corin-free RPMI for the remainder of 48 hours. (**C**) Mean ± SD intracellular EBV genome copy number from n=3 replicates of P3HR-1 or MUTU I cells treated with Corin for 48 hours. Blots are representative of n=3 independent replicates. *p<0.05, **p<0.01, ***p< 0.001.

Extended Data Figure S6. Corin induces EBV lytic reactivation in EBV+ cancer cells and xenograft tumors.
(**A**) Immunoblot analysis of WCL from Akata cells with stable GFP versus MYC cDNA overexpression that were treated with Corin for 48 hours, as indicated. (**B**) Immunoblot analysis of WCL from control or BZLF1 knockout (KO) Akata cells treated with Corin for 48 hours. (**C**) Immunoblot analysis of WCL from Akata, KEM I, EB3, Rael Burkitt cells, GM15892 LCLs or SNU-719 gastric carcinoma cells treated with Corin for 48 hours. (**D**) Immunoblot analysis of WCL from EBV+/KSHV+ BC-1 or KSHV+ BCBL-1 primary effusion lymphoma cells treated with Corin for 48 hours. (**E**) KSHV+ iSLK.219 epithelial cells with conditional doxycycline (Dox) inducible KSHV immediate early RTA expression was treated with DMSO, Dox (0.5 ug/ml) or Corin (5 μM) for 24 hours, and then maintained in growth media without Dox or Corin. 48 hours later, immunofluorescence analysis was performed for GFP (stably expressed from the KSHV genome) versus for red fluorescence protein (RFP, which reports KSHV lytic gene expression). Scale bar = 650 μm. (**F**) Quantification of BZLF1+ (upper panel) versus BMRF1+ (bottom panel) cells from xenograft tumors as in **Fig. 3I** from animals treated with vehicle versus Corin. Shown are the mean ± SEM BZLF1+ or BMRF1+ cells from four randomly selected fields per mouse were quantitated by Image J, using the Cell Counter plugin. (**G**) qPCR analysis of EBV immediate early BZLF1, early BMRF1 and late BLLF1 (encodes gp350) mRNA abundances in xenograft tumors from mice post-treatment with vehicle or Corin, as in **Fig. 3I**. (**H**) Intracellular EBV genome copy numbers, measured by qPCR, from xenograft tumors of mice post-treatment with vehicle or Corin, as in **Fig. 3I**. The horizontal lines indicate mean values. All blots and immunofluorescence images shown are representative of n=3 replicates.

Extended Data Figure S7. Analysis of LSD1 depletion effects on *oriLyt*^{R} H3K4 and H3K9 methylation.
(**A**) ChIP-qPCR analysis of H3K4me1, H3K4me2, and H3K4me3 levels in control vs LSD1, ZNF217 or CoREST depleted LCLs. Shown are mean ± SD ChIP-qPCR % input values from n=3 replicates of Akata cells expressing control, LSD1, ZNF217, or CoREST sgRNAs, followed by qPCR with primers specific for *oriLyt*^{R}. (**B**) Mean ± SD ChIP-qPCR % input values from n=3 replicates of Akata cells expressing control or LSD1 sgRNA, followed by qPCR with primers specific for the BZLF1 promoter or oriLyt regions. T-test was performed for same antibody ChIP between control and LSD1/ZNF217/CoREST depleted cells. NS: not significant; *p<0.05; **p < 0.01; ***p < 0.001.

Extended Data Figure S8. C12 or Corin effects on H3K4, H3K9 and H3K27 epigenetic marks at the *OriLyt* and *BZLF1* promoter regions.
(**A**) ChIP-qPCR analysis of LSD1 and ZNF217 occupancy in Akata cells 24 hours after treatment with vehicle, C12 (2.5 µM) or Corin (2.5 µM). Shown are mean ± SD ChIP-qPCR % input values from n=3 replicates. (**B**) ChIP-qPCR analysis as in (A) of H3K4me1, H3K4me2 and H3K4me3 abundances at the BZLF1 promoter or oriLyt regions. (**C**) ChIP-qPCR analysis as in (B) for H3K9Ac and H3K27Ac abundances at the *BZLF1* promoter or *oriLyt* regions. T-test was performed to cross-compare ChIP-qPCR values from C12 or Corin treated cells with corresponding values from control cells. **p < 0.01; ***p < 0.001; NS: not significant.

Extended Data Figure S9. Characterization of LSD1 KO, C12 and Corin effects on BZLF1 and OriLyt H3K9 and H3K27 epigenetic marks.
(**A**) ChIP-qPCR analysis of H3K9 and H3K27 acetylation levels in Akata cells expressing control versus independent LSD1 targeting sgRNAs. Shown are mean ± SD ChIP-qPCR % input values from n=3 replicates. (**B**) ChIP-qPCR analysis of H3K9me2 and H3K9me3 abundances at the *BZLF1* promoter or *oriLyt* regions in Akata cells treated with vehicle control, C12 (2.5 µM) or Corin (2.5 µM) for 24 hours. T-test was performed for same antibody ChIP between control and LSD1 depleted cells, or between control and C12 or Corin treated cells. *p < 0.05; **p < 0.01; NS: not significant.

Extended Data Figure S10. KMT2D supports Burkitt B-cell EBV lytic reactivation.
(**A**) Immunoblot analysis of WCL from Akata cells expressing control or KMT2D sgRNAs and that were mock-stimulated or stimulated for lytic reactivation by α-human IgG (10 µg/ml) cross-linking for 24 hours. (**B**) FACS analysis of PM gp350 signal on Akata cells expressing control or KMT2D sgRNA following 24 hours of mock-induction or induction of lytic reactivation by αIgG crosslinking as in (A). (**C**) Mean ± SD MFI PM gp350 values from n=3 replicates, as in (B). (**D**) qPCR analysis of intracellular EBV genome copy number in Akata cells expressing control or KMT2D sgRNA, treated with or without α-human IgG (10 µg/ml) for 24 hours. (**E**) Immunoblot analysis of WCL from Akata cells expressing control or KMT2D sgRNA and treated with vehicle or Corin (2.5 µM) for 24 hours. Blots are representative images of n = 3 replicates. **p < 0.01; ***p < 0.001.

Extended Data Table S1. Reagents, antibodies, and kits.

Extended Data Table S2. sgRNAs, plasmids, and primers.

Supplementary Files

This is a list of supplementary files associated with this preprint. Click to download.

- Table1.CRISPRCas9screenhits.xlsx
- Table2.CRISPRCas94HTNaBscreenhits.xlsx
- ExtendedDataTableS1.Reagentsantibodiesandkits.xlsx
- ExtendedDataTableS2.sgRNAsplasmidsandprimers.xlsx
- ExtendedFigures.pdf
